# Circulating GDF11 levels are decreased with age but are unchanged with obesity and type 2 diabetes

**DOI:** 10.18632/aging.101865

**Published:** 2019-03-21

**Authors:** Juan Añón-Hidalgo, Victoria Catalán, Amaia Rodríguez, Beatriz Ramírez, Camilo Silva, Juan C. Galofré, Javier Salvador, Gema Frühbeck, Javier Gómez-Ambrosi

**Affiliations:** 1 Metabolic Research Laboratory, Clínica Universidad de Navarra, Pamplona, Spain; 2 CIBER Fisiopatología de la Obesidad y Nutrición (CIBEROBN), Instituto de Salud Carlos III, Pamplona, Spain; 3 Obesity and Adipobiology Group, Instituto de Investigación Sanitaria de Navarra (IdiSNA), Pamplona, Spain; 4 Department of Endocrinology and Nutrition, Clínica Universidad de Navarra, Pamplona, Spain

**Keywords:** GDF11, aging, fat-free mass, obesity, type 2 diabetes

## Abstract

Growth differentiation factor 11 (GDF11) is a member of the transforming growth factor β (TGFβ) superfamily which declines with age and exerts anti-aging regenerative effects in skeletal muscle in mice. However, recent data in humans and mice are conflicting casting doubts about its true functional actions. The aim of the present study was to compare the circulating concentrations of GDF11 in individuals of different ages as well as body weight and glycemic status. Serum concentrations of GDF11 were measured by ELISA in 319 subjects. There was a significant increase in GDF11 concentrations in people in the 41-50 y group and a decline in the elder groups (61-70 and 71-80 y groups, *P*=0.008 for the comparison between all age groups). However, no significant correlation between fat-free mass index (FFMI), a formula used to estimate the amount of muscle mass in relation to height, and logGDF11 was observed (*r*=0.08, *P*=0.197). Moreover, no significant differences in circulating concentrations of GDF11 regarding obesity or glycemic status were found. Serum GDF11 concentrations in humans decrease in older ages being unaltered in obesity and T2D. Further studies should determine the exact pathophysiological role of GDF11 in aging.

## Introduction

Obesity prevalence has increased alarmingly threatening the health advances achieved in the last decades [1]. Importantly, obesity favors the development of comorbidities such as type 2 diabetes mellitus (T2D), cardiovascular disease, non-alcoholic fatty liver disease and cancer, leading to an increase in morbidity and mortality in relation to excess adiposity [1,2].

Body mass index (BMI) is the most frequently used tool for obesity diagnosis [3], but in spite of its wide use BMI is only a surrogate measure of body fatness and does not provide an accurate measure of body composition [4]. Noteworthy, obesity is defined as an excess accumulation of body fat, with the amount of this excess fat actually being responsible for most obesity-associated health risks [5]. A progressive increase in body adiposity is one of the features of aging [6]. This occurs even in individuals who maintain a constant BMI as they become older [7]. The observed discrepancies are emphasized after middle age and during the menopause in women. These data reflect that changes in body weight are not parallel to changes in body composition, with a continuous increase in adiposity, as well as a predisposition to accumulation in the visceral region [8,9], while a decrease in skeletal muscle mass takes place associated with an increased risk of disability [10].

The transforming growth factor (TGF)-β superfamily of signaling proteins consists of more than 30 members exerting pleiotropic functions by using common mechanisms of signal transduction [11-13]. In mammals, there are at least 3 subfamilies: TGF-βs, bone morphogenetic proteins (BMPs)/growth differentiation factors (GDFs) and activins/inhibins [12,13]. GDF11 is expressed in skeletal muscle, heart, intestine, pancreas, kidney, developing nervous system, olfactory system, as well as in the retina [14]. GDF11 reportedly declines in aging mice and promotes skeletal muscle regeneration increasing strength and endurance exercise capacity [15], at the same time as exerting anti-aging effects in heart [16] and brain [17]. However, further studies have found opposite results showing that GDF11 may produce no effect [18] or even skeletal muscle atrophy rather than regeneration [13,14] or inhibition of skeletal muscle growth [19]. In humans, results regarding circulating concentrations of GDF11 are conflicting showing a decrease [20], an increase or a trend to an increase [21-23], or no change [22,24] with aging.

We aimed to investigate in humans the relation of GDF11 with aging and its possible influence on the changes in body composition that take place with aging and the potential repercussion on metabolism. We studied the circulating concentrations of GDF11 in a cohort of subjects with different weight as well as glycemic status.

## RESULTS

**Figure 1:**
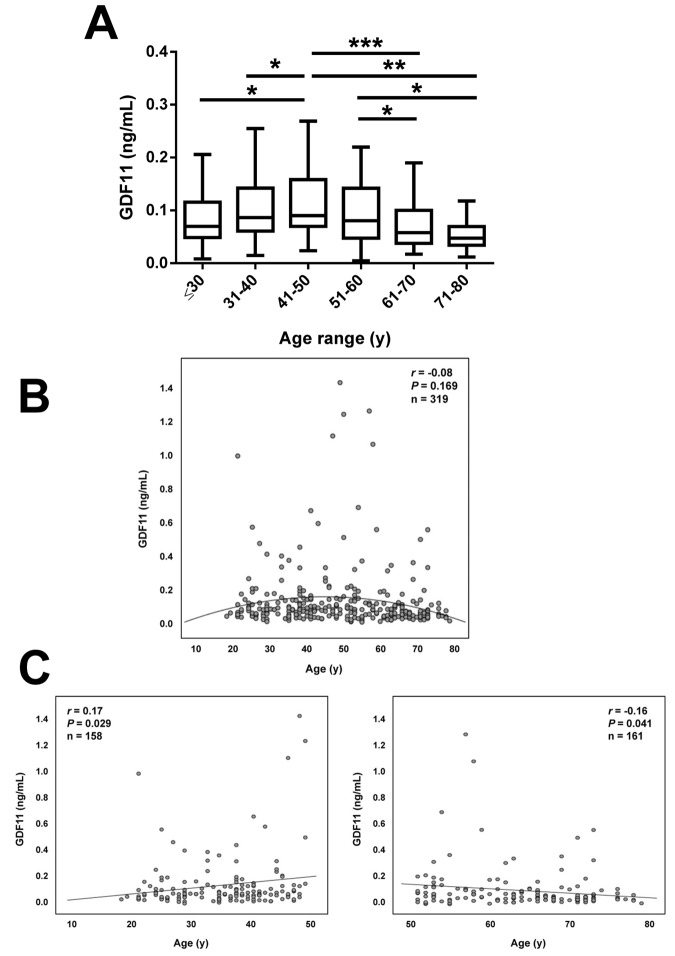
**Effect of aging on serum GDF11 concentrations.** (**A**) Comparison of serum GDF11 in the whole sample aged between 18 and 79 y segregated by decades (≤30 y, n=53), (31-40 y, n=54), (41-50 y, n=51), (51-60 y, n=54), (61-70 y, n=59), (71-80 y, n=48). Box represents interquartile range and median inside, with whiskers plotted according to the Tukey method. Statistical differences between groups were analyzed by one-way ANOVA followed by Fisher’s LSD tests. *P<0.05, **P<0.01 and ***P<0.001. (**B**) Scatter diagram showing the relationship between circulating concentrations of GDF11 and age. Pearson’s correlation coefficient and *P* value are indicated. The quadratic line of adjustment of data is shown. (**C**) Scatter diagrams showing the correlation between circulating concentrations of GDF11 and age in the subjects segregated by being below (left) or over (right) 50 years of age. Pearson’s correlation coefficients and *P* values are indicated.

**Figure 2:**
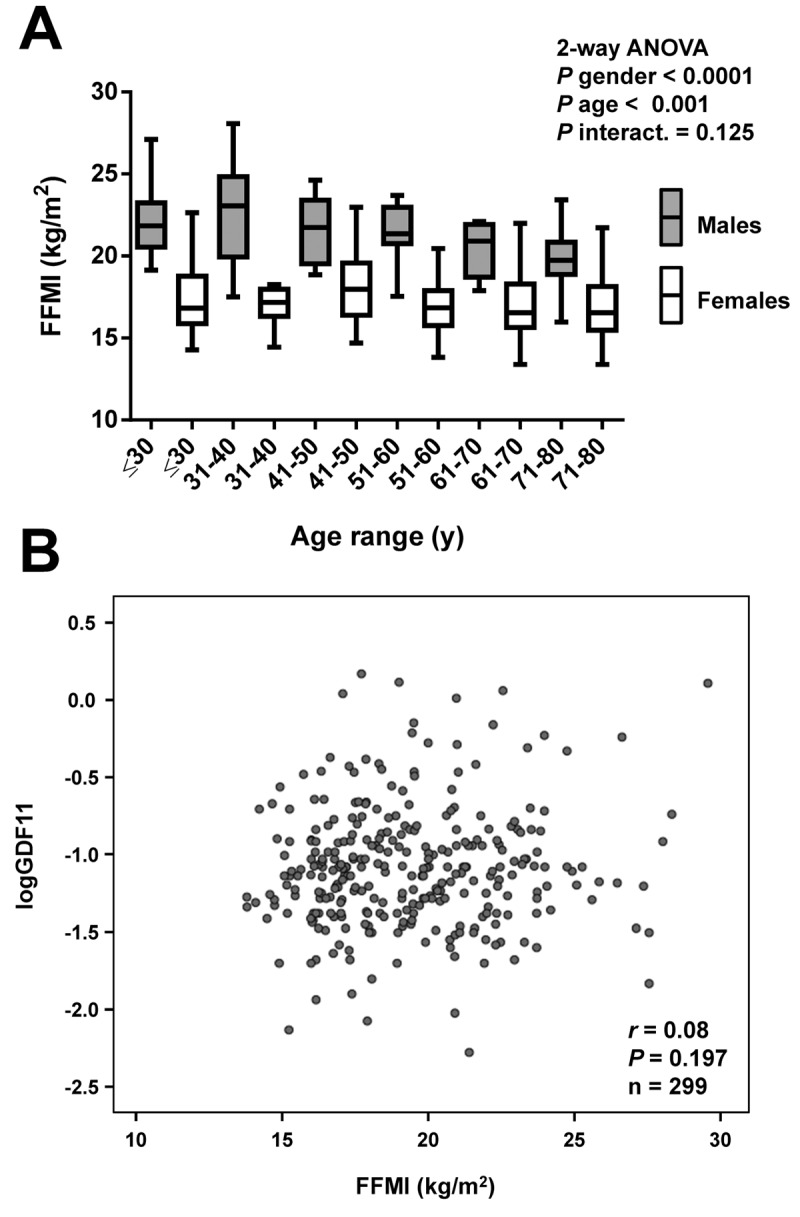
**Correlation of GDF11 levels with FFMI.** (**A**) Comparison of fat-free mass index (FFMI) in the whole sample aged between 18 and 79 y segregated by gender (males in grey and females in white) and decades (≤30 y, males n=18, females n=31), (31-40 y, males n=20, females n=34), (41-50 y, males n=24, females n=27), (51-60 y, males n=21, females n=33), (61-70 y, males n=14, females n=35), (71-80 y, males n=20, females n=22). Box represents interquartile range and median inside, with whiskers plotted according to the Tukey method. Statistical differences between groups were analyzed by two-way ANOVA. (**B**) Scatter diagram showing the relationship between circulating concentrations of GDF11 and FFMI. Pearson’s correlation coefficient and *P* value are indicated.

Anthropometric and biochemical characteristics of the individuals included in the study are shown in Table 1. From the whole cohort, 126 (39%) were males and 193 (61%) were females. We measured serum GDF11 concentrations in our sample aged between 18 and 79 y. Significant differences in GDF11 levels were observed when the data were segregated by decades (*P*=0.008, Figure 1A). A significant increase in the 41-50 y group was observed. Circulating concentrations of GDF11 progressively declined thereafter with significant decreases observed in the 61-70 and the 71-80 y groups. In agreement with this pattern across decades of age, we found no linear correlation of GDF11 levels with age (*r*=-0.08; *P*=0.169), with data following a quadratic distribution (Figure 1B). However, we observed a significant negative correlation of logGDF11 with age (*r*=-0.16; *P*=0.005) (Table 2) and when we segregated the subjects by being below or over 50 years of age GDF11 levels correlated positively (*r*=0.17 ; *P*=0.029) and negatively (*r*=-0.16; *P*=0.041) with age in individuals below and over 50, respectively (Figure 1C). We repeated the analysis with logarithmically transformed data to reduce dispersion obtaining an even higher *P* value in the ANOVA (*P*<0.001, data not shown). Moreover, after the removal of the 5 data higher than 1.0 ng/mL the differences between groups were still significant (*P*=0.005). Interestingly, without those 5 points we found a global negative correlation of GDF11 levels with age (*r*=-0.14, *P*=0.014, n=314), although the correlation taking into consideration only those subjects with age ≥ 50 was marginal (*r*=-0.14, *P*=0.087, n=159). FFMI, a formula used to estimate the amount of muscle mass in relation to height, was significantly decreased with age (*P*<0.001), something that was more marked in men than in women (*P*<0.0001) (Figure 2A). However, no significant correlation between FFMI and logGDF11 was observed (*r*=0.08, *P*=0.197) (Figure 2B and Table 2). Since the effect of age on FFMI was more evident in males than in females, we analyzed the correlation of GDF11 with age splitting the sample according to gender. We found a significant negative correlation of logGDF11 with age in males (*r*=-0.21, *P*=0.019) and a marginal one in females (*r*=-0.12, *P*=0.085). After removing the 5 data higher than 1.0 ng/mL both negative correlations were significant (*r*=-0.19, *P*=0.040 for males and *r*=-0.15, *P*=0.038 for females).

**Table 1:** Demographic and biochemical characteristics of the individuals at enrollment.

	**All**	**Male**	**Female**	***P***
n	319	126	193	
Age, y	50 ± 16	50 ± 17	50 ± 16	0.871
Weight, kg	98 ± 28	115 ± 27	86 ± 23	<0.001
BMI, kg/m^2^	35.1 ± 8.8	37.8 ± 8.3	33.4 ± 8.7	<0.001
Body fat, %*	44.0 ± 9.6	41.1 ± 8.7	46.0 ± 9.7	<0.001
FFM, kg*	53.2 ± 12.6	65.6 ± 9.2	45.2 ± 6.7	<0.001
FFMI, kg/m^2^*	19.0 ± 3.1	21.6 ± 2.5	17.3 ± 2.1	<0.001
Waist circumference, cm	111 ± 21	122 ± 18	103 ± 19	<0.001
SBP, mm Hg	126 ± 18	132 ± 16	122 ± 18	<0.001
DBP, mm Hg	78 ± 11	82 ± 11	75 ± 9	<0.001
Glucose, mg/dL	102± 22	108 ± 24	98 ± 20	<0.001
Insulin, μU/mL	14.9 ± 13.6	18.1 ± 11.5	12.8 ± 14.5	<0.001
Glucose 2-h OGTT, mg/dL#	154 ± 59	161 ± 56	150 ± 61	0.158
Insulin 2-h OGTT, mg/dL#	124 ± 79	135 ± 78	117 ± 80	0.087
HOMA	4.0 ± 4.3	5.2 ± 4.4	3.3 ± 4.0	<0.001
QUICKI	0.34 ± 0.05	0.32 ± 0.04	0.35 ±0.05	<0.001
Triglycerides, mg/dL	115 ± 68	134 ± 65	103 ± 67	<0.001
Cholesterol, mg/dL	194 ± 40	190 ± 46	197 ± 35	0.182
LDL-cholesterol, mg/dL	117 ± 37	118 ± 43	116 ± 33	0.651
HDL-cholesterol, mg/dL	54.8 ± 17.1	45.9 ± 10.9	60.7 ± 17.9	<0.001
Uric acid, mg/dL	5.6 ± 1.7	6.8 ± 1.4	4.7 ± 1.3	<0.001
CRP, mg/L	7.8 ± 7.9	7.2 ± 8.4	8.2 ± 7.6	0.820
WBC, 10^6^ cells/mL	6.7 ± 2.0	6.8 ± 1.8	6.6 ± 2.1	0.471
ALT, U/L	23 ± 15	30 ± 17	19 ± 12	<0.001
AST, U/L	18 ± 8	20 ± 8	16 ± 7	<0.001
AST/ALT ratio	0.89 ± 0.32	0.76 ± 0.28	0.98 ± 0.31	<0.001
γ-GT, U/L	28 ± 32	37 ± 42	22 ± 21	<0.001
Creatinine, mg/dL	0.82 ± 0.22	0.97 ± 0.21	0.72 ± 0.15	<0.001
Leptin, ng/mL	34.8 ± 27.7	25.2 ± 16.6	40.7 ± 31.4	<0.001
GDF11, ng/mL	0.126 ± 0.185	0.129 ± 0.197	0.124 ± 0.178	0.807

Data presented as mean ± SD. *Body composition was available in 299 subjects (117 males and 182 females). #OGTTs were available in 226 individuals (87 males and 139 females).

BMI, body mass index; FFM, fat-free mass; FFMI, fat-free mass index; SBP, systolic blood pressure; DBP, diastolic blood pressure; OGTT, oral glucose tolerance test; HOMA, homeostatic model of assessment; QUICKI, quantitative insulin sensitivity check index; CRP, C-reactive protein; WBC, white blood cells; ALT, alanine aminotransferase; AST, aspartate aminotransferase; γ-GT, γ-glutamyltransferase. Differences between groups were analyzed by two-tailed unpaired Student’s t tests. CRP concentrations were logarithmically transformed for statistical analysis.

**Table 2:** Univariate analysis of the correlation between GDF11 and other variables, unadjusted and after adjusting for age.

	**Serum logGDF11**			
	Unadjusted correlation		Adjusted correlation	
Variable	*r*	*P* value	*r*	*P* value
Sex	0.01	0.810	0.01	0.828
Age	**-0.16**	**0.005**	—	—
Weight	0.11	0.059	0.08	0.168
BMI	**0.12**	**0.040**	0.11	0.053
Body fat	0.07	0.189	0.09	0.096
FFM	0.06	0.308	0.02	0.769
FFMI	0.08	0.197	0.06	0.347
Waist circumference	0.10	0.076	0.11	0.055
SBP	0.01	0.902	0.06	0.330
DBP	0.04	0.452	0.06	0.304
Glucose	0.01	0.923	0.04	0.461
Insulin	0.08	0.160	0.09	0.142
Glucose 2 h OGTT	0.02	0.776	0.05	0.425
Insulin 2 h OGTT	-0.06	0.360	-0.03	0.659
HOMA	0.08	0.193	0.08	0.152
QUICKI	-0.04	0.484	-0.07	0.243
Triglycerides	0.03	0.578	0.05	0.437
Cholesterol	0.06	0.315	0.08	0.178
LDL-cholesterol	0.07	0.252	0.08	0.184
HDL-cholesterol	-0.04	0.466	-0.03	0.604
Uric acid	0.01	0.823	0.01	0.824
logCRP	0.13	0.106	0.12	0.144
WBC	-0.02	0.744	-0.03	0.658
ALT	0.09	0.130	0.07	0.197
AST	-0.01	0.812	-0.01	0.887
AST/ALT ratio	**-0.12**	**0.044**	-0.10	0.084
γ-GT	-0.01	0.846	0.01	0.922
Creatinine	0.05	0.387	0.08	0.182
Leptin	0.11	0.076	0.11	0.062

Values are Pearson’s correlation coefficients and associated *P* values. CRP concentrations were logarithmically transformed for statistical analysis. BMI, body mass index; FFM, fat-free mass; FFMI, fat-free mass index; SBP, systolic blood pressure; DBP, diastolic blood pressure; OGTT, oral glucose tolerance test; HOMA, homeostatic model assessment; QUICKI, quantitative insulin sensitivity check index; CRP, C-reactive protein; WBC, white blood cells; ALT, alanine aminotransferase; AST, aspartate aminotransferase; γ-GT, γ-glutamyltransferase. For correlation with gender, male=1 and female=2 was used.

**Figure 3:**
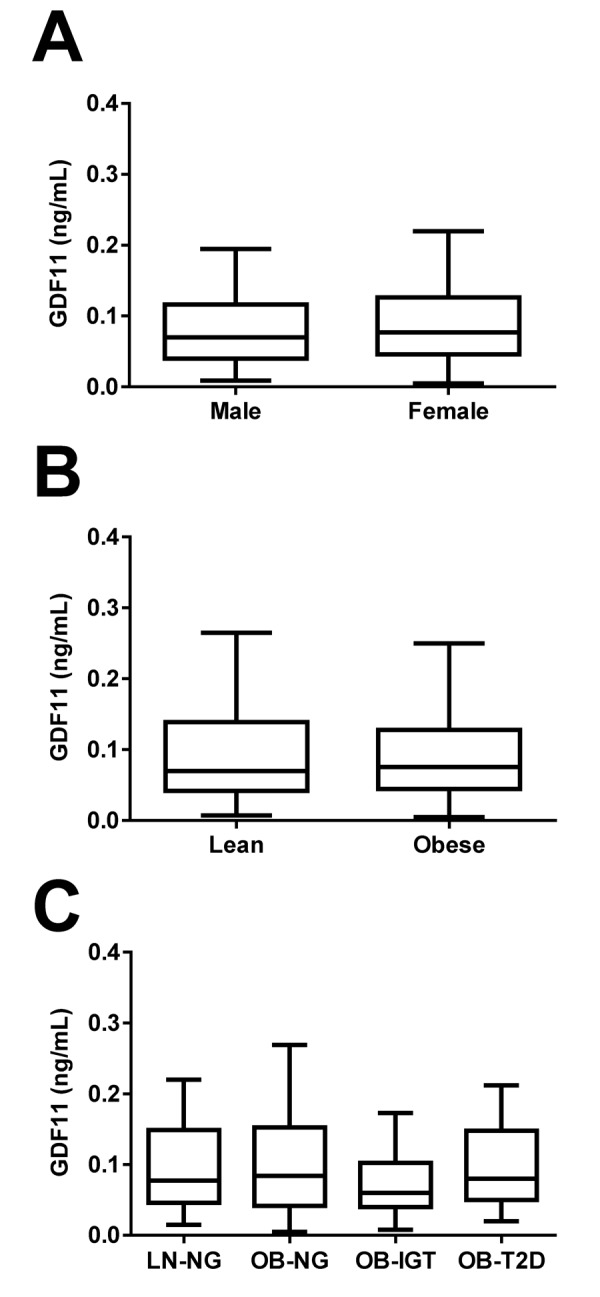
**Effect of gender, obesity and type 2 diabetes on GDF11 levels.** (**A**) Comparison of serum GDF11 concentrations between males (n=126) and females (n=193). Statistical difference was analyzed by two-tailed unpaired Student’s t test. (**B**) Comparison of serum GDF11 concentrations between lean (n=49) and obese (n=228) individuals. Statistical difference was analyzed by two-tailed unpaired Student’s t test. (**C**) Serum GDF11 concentrations in the lean normoglycemic (LN-NG, n=40), obese normoglycemic (OB-NG, n=93), obese with impaired glucose tolerance (OB-IGT, n=46), and obese with type 2 diabetes (OB-T2D, n=47) groups of the whole sample. Statistical differences between groups were analyzed by one-way ANOVA. For the three figures, box represents interquartile range and median inside, with whiskers plotted according to the Tukey method.

No significant differences in circulating concentrations of GDF11 regarding gender (males 0.129 ± 0.197, females 0.124 ± 0.178 ng/mL; *P*=0.807) were found (Figure 3A). The study included 49 lean subjects (7 males and 42 females) and 228 (109 males and 119 females) obese individuals classified according to BMI. No significant differences in GDF11 levels regarding obesity (lean 0.125 ± 0.170, obese 0.135 ± 0.202 ng/mL; *P*=0.757) were observed (Figure 3B). All obese groups showed altered body fat, waist circumference, blood pressure, lipid and inflammatory profiles, as well as hepatic enzymes as compared to the lean group (*P*<0.05 for all) (Table 3). Moreover, the obese groups with glucose intolerance or T2D exhibited, as expected, increased glucose and insulin levels 2 h after the OGTT (*P*<0.05 for both). Furthermore, the T2D group showed higher (*P*<0.05) waist circumference, SBP and DBP, glucose metabolism variables, LDL-C, uric acid and hepatic enzymes, than the other groups. Noteworthy, circulating concentrations of GDF11 were unchanged regarding glycemic status [LN-NG (n=40) 0.138 ± 0.183, OB-NG (n=93) 0.128 ± 0.136, OB-IGT (n=46) 0.145 ± 0.276, OB-T2D (n=47) 0.162 ± 0.251 ng/mL; *P*=0.834] as depicted in Figure 3C. We repeated the analysis regarding gender, obesity and T2D segregating the subjects by being below or over 50 years of age finding similar results (data not shown). Interestingly, circulating concentrations of GDF11 showed a slight correlation with BMI (*r*=0.12, *P*=0.040) that was lost after adjustment by age (*r*=0.11, *P*=0.053). We found no correlation with any other anthropometric variable including body fat percentage (*r*=0.07, *P*=0.189), waist circumference (*r*=0.10, *P*=0.076), FFM (*r*=0.06, *P*=0.308) or FFMI (*r*=0.08, *P*=0.197) (Table 2). Finally, we found a significant negative correlation of GDF11 levels with the AST/ALT ratio (*r*=-0.12, *P*=0.044) that was lost after adjustment by age (*r*=-0.10, *P*=0.084).

**Table 3:** Demographic and biochemical characteristics of the study sample classified according to obesity and glycemic status.

	**LN-NG**	**OB-NG**	**OB-IGT**	**OB-T2D**	***P***
n	40	93	46	47	
Sex, M/F	7/33	37/56	21/25	22/25	0.020
Age, y	44 ± 15	49 ± 15	50 ± 16	51 ± 11	0.121
Weight, kg	66 ± 9 1010	109 ± 27*	100 ± 23*	117 ± 27*‡	<0.001
BMI, kg/m^2^	23.6 ± 1.1	39.2 ± 7.8*	36.8 ± 6.5*	42.0 ± 7.7*†‡	<0.001
Body fat, %	29.8 ± 6.3	48.6 ± 7.3*	47.2± 6.2*	49.3 ± 7.7*	<0.001
FFM, kg	47.0 ± 9.4	55.0 ± 12.6*	52.9± 12.8*	57.9 ± 13.4*	<0.001
FFMI, kg/m^2^	16.6 ± 1.7	19.8 ± 2.9*	19.3 ± 3.1*	20.8 ± 2.8*†‡	<0.001
Waist circumference, cm	84 ± 8 1010	119 ± 17*	115 ± 16*	126 ± 16*†‡	<0.001
SBP, mm Hg	114 ± 18	127 ± 17*	125 ± 13*	134 ± 16*†‡	<0.001
DBP, mm Hg	69 ± 7	80 ± 10*	77 ± 8*	83 ± 12*‡	<0.001
Glucose, mg/dL	89 ± 15	95 ± 10*	101 ± 11*†	120± 22*†‡	<0.001
Insulin, μU/mL	4.6 ± 3.6	13.8 ± 9.1*	16.7 ± 12.0*	25.0 ± 21.8*†‡	<0.001
Glucose 2 h OGTT, mg/dL	94 ± 25	113 ± 18*	164 ± 18*†	245 ± 38*†‡	<0.001
Insulin 2 h OGTT, mg/dL	56 ± 17	95 ± 64	160 ± 77*†	167 ± 77*†	<0.001
HOMA	1.0 ± 0.9	3.3 ± 2.2*	4.3 ± 3.2*	7.4 ± 6.5*†‡	<0.001
QUICKI	0.40 ± 0.05	0.33 ± 0.04*	0.33 ± 0.04*	0.30 ± 0.03*†‡	<0.001
Triglycerides, mg/dL	68 ± 29	111 ± 56*	118 ± 46*	149 ± 60*†‡	<0.001
Cholesterol, mg/dL	180 ± 34	199 ± 39*	204 ± 44*	200 ± 33*	0.017
LDL-cholesterol, mg/dL	96 ± 30	124 ± 35*	126 ± 42*	125 ± 32*	<0.001
HDL-cholesterol, mg/dL	70 ± 17	53 ± 16*	56 ± 21*	45 ± 11*†‡	<0.001
Uric acid, mg/dL	4.0 ± 1.4	5.7 ± 1.4*	5.8 ± 1.7*	6.6 ± 1.8*†‡	<0.001
CRP, mg/L	1.6 ± 3.3	9.8 ± 8.4*	7.4 ± 6.5*	10.1 ± 10.0*	<0.001
WBC, 10^6^ cells/mL	5.3 ± 1.2	6.8 ± 2.0*	6.5 ± 1.3*	8.1 ± 2.6*†‡	<0.001
ALT, U/L	18 ± 10	22 ± 16	24 ± 12	33 ± 20*†‡	<0.001
AST, U/L	18 ± 8	17 ± 7	17 ± 7	21 ± 10**†‡**	0.042
AST/ALT ratio	1.10 ± 0.27	0.89 ± 0.32*	0.77 ± 0.19*†	0.73 ± 0.27*†	<0.001
γ-GT, U/L	16 ± 14	29 ± 40*	26 ± 17	44 ± 30*†‡	<0.001
Creatinine, mg/dL	0.74 ± 0.13	0.82 ± 0.20	0.84 ± 0.25	0.83 ± 0.21	0.093
Leptin, ng/mL	12.5 ± 20.3	44.4 ± 26.0*	43.0 ± 32.5*	42.7 ± 30.3*	<0.001

Data presented as mean ± SD. Individuals were matched by age and sex in the whole sample, and by body fat in the obese groups. BMI, body mass index; FFM, fat-free mass; FFMI, fat-free mass index; SBP, systolic blood pressure; DBP, diastolic blood pressure; OGTT, oral glucose tolerance test; HOMA, homeostatic model assessment; QUICKI, quantitative insulin sensitivity check index; CRP, C-reactive protein; WBC, white blood cells; ALT, alanine aminotransferase; AST, aspartate aminotransferase; γ-GT, γ-glutamyltransferase. Differences between groups were analyzed by ANOVA followed by LSD tests. *P<0.05 vs LN-NG, **†***P*<0.05 vs OB-NG and **‡***P*<0.05 vs OB-IGT. Differences in gender distribution were analyzed by χ^2^ analysis. CRP concentrations were logarithmically transformed for statistical analysis.

## DISCUSSION

The main findings of the present study are that 1) serum concentrations of GDF11 peaks at 41-50 y of age progressively declining thereafter with significant decreases observed in the 61-70 and the 71-80 y groups, although this decline seems to have no translation in a reduced FFM; and 2) GDF11 levels showed no gender dimorphism and were unchanged due to obesity or glycemic status.

GDF11 has been shown to decline with aging in mice [15,16,25,26] and other species [25]. In humans, data regarding circulating levels of GDF11 are conflicting showing a decrease [20,27,28], an increase or a trend towards an increase [21-23,29] or no change [22,24,28] with aging. In the present study, the decline in serum GDF11 levels observed in older age was independent of gender and we did not observe differences between genders in GDF11 levels in agreement with previous studies in mice [26] and humans [20]. The curvilinear relationship of circulating GDF11 concentrations with age suggests that there is a deficit in GDF11 actions at middle age that is compensated by a hypersecretion that fails at advanced ages, above the 60’s. Further larger mechanistic studies will prove whether or not there is a decline with aging in humans and the exact physiological functions in which circulating GDF11 is involved in. GDF11 has been reported to exert anti-aging effects promoting skeletal muscle [15], heart [16] and brain [17] regeneration in mice. However, other studies have shown opposite results suggesting that GDF11 may produce no effect [18] or skeletal muscle atrophy rather than regeneration [13,14]. Interestingly, a recent study has shown no relationship of GDF11 with skeletal muscle strength in humans [23]. Moreover, a previous work suggests that high GDF11 levels predict longer life span [26], while another study reported that GDF11 administration does not extend lifespan in a mouse model of premature aging [30]. In the present study we found no relationship between circulating concentrations of GDF11 and FFM or FFMI in a group of subject encompassing people from ages between 18 and 79 y pointing out to a lack of effect of GDF11 in the regulation of skeletal muscle mass in humans. However, a previous study has shown that individuals with higher GDF11 are more likely to exhibit frailty, but skeletal muscle mass in that study was not measured and the influence of other factors in the presence of frailty in addition to sarcopenia cannot be discarded [24].

Aging is characterized by a progressive increase in body adiposity that may be concomitantly accompanied by a decrease in muscle mass associated with an increased risk of morbidity and mortality [6,10,31]. In the present study we did not observe differences in serum GDF11 concentrations in obese subjects as compared to lean ones, or a clear correlation of GDF11 levels with BMI or body adiposity. This is to our knowledge the first study analyzing the levels of GDF11 in obesity. In a previous study, no significant differences in BMI were observed in a smaller sample of subjects segregated by serum GDF11 tertiles [24]. Another study has reported slight but significantly lower levels of GDF11/8 in overweight individuals [27]. Interestingly, it has been reported that serum GDF11/8 levels [27] and GDF11 expression in skeletal muscle [32] are decreased in mice fed a high-fat diet and that exogenously administered GDF11 reduces body weight in both young and old mice [25]. Larger and intervention studies are needed to fully clarify whether GDF11 may play a role in weight and adiposity regulation in humans.

We analyzed the levels of serum GDF11 in obese subjects with different glycemic status to study whether GDF11 may be involved in IGT and T2D. No significant differences in obese patients with IGT or T2D as compared to obese or lean normoglycemic subjects were observed. A previous study analyzing the levels of GDF11 in human T2D found increased levels of GDF11 only in T2D patients with cardiovascular disease, but not in T2D patients free of cardiovascular problems [28]. Contrary to our results, another report showed that increased circulating GDF11 was detected in a greater proportion of subjects with diabetes [24]. It has been reported that incubation with GDF11 does not ameliorate palmitate-induced insulin resistance in murine myotubes [32]. On the other hand, it has been reported that adeno-associated viruses-GDF11 improve glucose tolerance in apoE^-/-^ mice, which could be in relation to the endothelium-protective and antiatherosclerotic effects of GDF11 [27]. Moreover, exogenous GDF11 attenuates the development of T2D in mice through the improvement of β-cell function and survival via the activation of TGF-β/Smad2 and PI3K-AKT-FoxO1 [33]. Intervention and genetic studies analyzing the direct effect of GDF11 or the effect of genetic alterations in the GDF11 locus will help to clarify the exact role of GDF11 in glucose homeostasis in humans.

We observed a general lack of correlation for GDF11 with the anthropometric and metabolic variables studied with the exception of age, BMI, and the AST/ALT ratio (Table 2). The association of circulating concentrations of GDF11 with the AST/ALT ratio suggests that this factor may be involved in the development of non-alcoholic fatty liver disease. Further studies aimed to assess the role of GDF11 in hepatic lipid accumulation will clarify this finding.

Some potential limitations of our study should be pointed out. First, our study was conducted in Caucasian subjects and it would need to be determined whether our findings regarding GDF11 levels extend to other populations. Second, we did not measure skeletal muscle mass directly and used instead FFM and FFMI. Future studies measuring skeletal muscle mass directly may shed more light on the role of GDF11 in skeletal muscle regulation.

In conclusion, our results show that circulating concentrations of GDF11 are decreased in elderly subjects (over 60 y) without apparent influence on FFM. Moreover, GDF11 levels are not modified in obesity or T2D. Further studies modulating GDF11 levels and activity will undoubtedly help to elucidate the exact role of GDF11 in aging and whether it plays a role in skeletal muscle mass regulation in humans.

## MATERIALS AND METHODS

### Study population

We conducted a cross-sectional analysis of 319 patients (126 men and 193 women) aged 18-79 years, with similar socio-economical characteristics, including patients visiting the Department of Endocrinology and Nutrition of the Clínica Universidad de Navarra (Pamplona, Spain) for weight loss treatment as well as hospital and University staff undergoing an annual routine health check-up. Normal weight was considered as having a body mass index (BMI)<25 kg/m^2^, while obesity was defined as having a BMI≥30 kg/m^2^ following the World Health Organization criteria [4]. Subjects were additionally classified as having normoglycemia (NG), impaired glucose tolerance (IGT) or type 2 diabetes (T2D) following the criteria of the American Diabetes Association [34] based on plasma glucose 2 h after an oral glucose tolerance test (OGTT) in the 226 individuals with an available OGTT. Glucose intolerance or T2D was of recent-onset being diagnosed after the OGTT and, therefore, participants were not on antidiabetic medication or insulin therapy. Participants underwent a clinical assessment including medical history, physical examination, body composition analysis and comorbidity evaluation performed by a multidisciplinary consultation team. Individuals with signs of infection were excluded. The experimental design was approved by the Research Ethics Committee of the University of Navarra (protocol 2017.121) and the study was performed in accordance with the ethical standards as laid down in the Declaration of Helsinki and its later amendments. Volunteers gave their written informed consent to participate in the study.

### Anthropometry

The anthropometric and body composition determinations as well as the blood extraction were performed on a single day. Height was measured to the nearest 0.1 cm with a Holtain stadiometer (Holtain Ltd., Crymych, UK), while body weight was measured with a calibrated electronic scale to the nearest 0.1 kg with subjects wearing a swimming suit and cap. BMI was calculated as weight in kg divided by the square of height in meters. Waist circumference was measured at the midpoint between the iliac crest and the rib cage on the midaxillary line. Blood pressure was measured after a 5-minute rest in the semi-sitting position with a sphygmomanometer. Blood pressure was determined at least 3 times at the right upper arm and the mean was used in the analyses. Body density was estimated by air displacement plethysmography (Bod-Pod^®^, Life Measurements, Concord, CA). Percentage of body fat was estimated from body density using the Siri equation as previously described [4]. Fat-free mass (FFM) index (FFMI) was calculated as FFM in kg divided by the square of height in meters [35]. Twenty subjects out of 319 had missing body composition analysis.

### Blood biochemistry

Blood samples were collected after an overnight fast in the morning in order to avoid potential confounding influences due to hormonal rhythmicity. Plasma glucose was analyzed by an automated analyzer (Roche/Hitachi Modular P800) as previously described [36]. Insulin was measured by means of enzyme-amplified chemiluminescence assay (Immulite 2000, Siemens AG, Erlangen, Germany). Indirect measures of insulin resistance and insulin sensitivity were calculated by using the homeostatic model assessment (HOMA) and the quantitative insulin sensitivity check index (QUICKI), respectively. Total cholesterol and triglyceride concentrations were determined by enzymatic spectrophotometric methods (Roche, Basel, Switzerland). Serum HDL-C was quantified by a colorimetric method in a Beckman Synchron^®^ CX analyzer (Beckman Instruments, Ltd., Bucks, UK). Low-density lipoprotein (LDL-C) was calculated by the Friedewald formula. High-sensitivity C-reactive protein (CRP) was measured using the Tina-quant CRP (Latex) ultrasensitive assay (Roche). White blood cell (WBC) count was measured using an automated cell counter (Beckman Coulter, Fullerton, CA). Uric acid, alanine aminotransferase (ALT), aspartate aminotransferase (AST), γ-glutamyltransferase (γ-GT), and creatinine were measured by enzymatic tests (Roche) in an automated analyzer (Roche/Hitachi Modular P800). The AST/ALT ratio was calculated as an indirect indicator of hepatic steatosis and fatty liver disease [37]. Leptin levels were quantified by a double-antibody RIA method (Linco Research, Inc., St. Charles, MO) as previously described [38,39]; intra-and inter-assay coefficients of variation were 5.0% and 4.5%, respectively. Serum GDF11 concentrations were determined using a validated ELISA kit (Human GDF11 ELISA kit, E01G0124, BlueGene Biotech, Shanghai, China) with intra-and inter-assay coefficients of variation being 5.5% and 7.8%, respectively. According to the manufacturer, no cross reactivity has been observed with any other analogue.

### Statistical analysis

Data are presented as mean ± SD unless otherwise indicated. Differences between groups were analyzed by one-way ANOVA followed by Fisher’s LSD tests or two-tailed unpaired Student’s t tests, as appropriate. Correlations between two variables were computed by Pearson’s correlation coefficients (*r*). The calculations were performed using SPSS 23 (SPSS, Chicago, IL) and GraphPad Prism 6 (GraphPad Software, Inc., La Jolla, CA). A *P* value lower than 0.05 was considered statistically significant.

## References

[1] Bray GA, Frühbeck G, Ryan DH, Wilding JP. Management of obesity. Lancet. 2016; 387:1947–56. 10.1016/S0140-6736(16)00271-3 26868660

[2] Afshin A, Forouzanfar MH, Reitsma MB, Sur P, Estep K, Lee A, Marczak L, Mokdad AH, Moradi-Lakeh M, Naghavi M, Salama JS, Vos T, Abate KH, et al, and GBD 2015 Obesity Collaborators. The GBD 2015 Obesity Collaborators. Health effects of overweight and obesity in 195 countries over 25 years. N Engl J Med. 2017; 377:13–27. 10.1056/NEJMoa1614362 28604169 PMC5477817

[3] Flegal KM, Shepherd JA, Looker AC, Graubard BI, Borrud LG, Ogden CL, Harris TB, Everhart JE, Schenker N. Comparisons of percentage body fat, body mass index, waist circumference, and waist-stature ratio in adults. Am J Clin Nutr. 2009; 89:500–08. 10.3945/ajcn.2008.26847 19116329 PMC2647766

[4] Gómez-Ambrosi J, Silva C, Galofré JC, Escalada J, Santos S, Millán D, Vila N, Ibañez P, Gil MJ, Valentí V, Rotellar F, Ramírez B, Salvador J, Frühbeck G. Body mass index classification misses subjects with increased cardiometabolic risk factors related to elevated adiposity. Int J Obes. 2012; 36:286–94. 10.1038/ijo.2011.100 21587201

[5] Heymsfield SB, Wadden TA. Mechanisms, pathophysiology, and management of obesity. N Engl J Med. 2017; 376:254–66. 10.1056/NEJMra1514009 28099824

[6] Kuk JL, Saunders TJ, Davidson LE, Ross R. Age-related changes in total and regional fat distribution. Ageing Res Rev. 2009; 8:339–48. 10.1016/j.arr.2009.06.001 19576300

[7] Prentice AM, Jebb SA. Beyond body mass index. Obes Rev. 2001; 2:141–47. 10.1046/j.1467-789x.2001.00031.x 12120099

[8] Hughes VA, Frontera WR, Roubenoff R, Evans WJ, Singh MA. Longitudinal changes in body composition in older men and women: role of body weight change and physical activity. Am J Clin Nutr. 2002; 76:473–81. 10.1093/ajcn/76.2.473 12145025

[9] Pararasa C, Bailey CJ, Griffiths HR. Ageing, adipose tissue, fatty acids and inflammation. Biogerontology. 2015; 16:235–48. 10.1007/s10522-014-9536-x 25367746

[10] Fantin F, Di Francesco V, Fontana G, Zivelonghi A, Bissoli L, Zoico E, Rossi A, Micciolo R, Bosello O, Zamboni M. Longitudinal body composition changes in old men and women: interrelationships with worsening disability. J Gerontol A Biol Sci Med Sci. 2007; 62:1375–81. 10.1093/gerona/62.12.1375 18166688

[11] Horbelt D, Denkis A, Knaus P. A portrait of Transforming Growth Factor β superfamily signalling: background matters. Int J Biochem Cell Biol. 2012; 44:469–74. 10.1016/j.biocel.2011.12.013 22226817

[12] Fan X, Gaur U, Sun L, Yang D, Yang M. The Growth Differentiation Factor 11 (GDF11) and Myostatin (MSTN) in tissue specific aging. Mech Ageing Dev. 2017; 164:108–12. 10.1016/j.mad.2017.04.009 28472635

[13] Hammers DW, Merscham-Banda M, Hsiao JY, Engst S, Hartman JJ, Sweeney HL. Supraphysiological levels of GDF11 induce striated muscle atrophy. EMBO Mol Med. 2017; 9:531–44. 10.15252/emmm.201607231 28270449 PMC5376753

[14] Harper SC, Brack A, MacDonnell S, Franti M, Olwin BB, Bailey BA, Rudnicki MA, Houser SR. Is growth differentiation factor 11 a realistic therapeutic for aging-dependent muscle defects? Circ Res. 2016; 118:1143–50. 10.1161/CIRCRESAHA.116.307962 27034276 PMC4829942

[15] Sinha M, Jang YC, Oh J, Khong D, Wu EY, Manohar R, Miller C, Regalado SG, Loffredo FS, Pancoast JR, Hirshman MF, Lebowitz J, Shadrach JL, et al. Restoring systemic GDF11 levels reverses age-related dysfunction in mouse skeletal muscle. Science. 2014; 344:649–52. 10.1126/science.1251152 24797481 PMC4104429

[16] Loffredo FS, Steinhauser ML, Jay SM, Gannon J, Pancoast JR, Yalamanchi P, Sinha M, Dall’Osso C, Khong D, Shadrach JL, Miller CM, Singer BS, Stewart A, et al. Growth differentiation factor 11 is a circulating factor that reverses age-related cardiac hypertrophy. Cell. 2013; 153:828–39. 10.1016/j.cell.2013.04.015 23663781 PMC3677132

[17] Katsimpardi L, Litterman NK, Schein PA, Miller CM, Loffredo FS, Wojtkiewicz GR, Chen JW, Lee RT, Wagers AJ, Rubin LL. Vascular and neurogenic rejuvenation of the aging mouse brain by young systemic factors. Science. 2014; 344:630–34. 10.1126/science.1251141 24797482 PMC4123747

[18] Smith SC, Zhang X, Zhang X, Gross P, Starosta T, Mohsin S, Franti M, Gupta P, Hayes D, Myzithras M, Kahn J, Tanner J, Weldon SM, et al. GDF11 does not rescue aging-related pathological hypertrophy. Circ Res. 2015; 117:926–32. 10.1161/CIRCRESAHA.115.307527 26383970 PMC4636963

[19] Jin Q, Qiao C, Li J, Li J, Xiao X. Neonatal systemic AAV-mediated gene delivery of GDF11 inhibits skeletal muscle growth. Mol Ther. 2018; 26:1109–17. 10.1016/j.ymthe.2018.01.016 29503194 PMC6079557

[20] Olson KA, Beatty AL, Heidecker B, Regan MC, Brody EN, Foreman T, Kato S, Mehler RE, Singer BS, Hveem K, Dalen H, Sterling DG, Lawn RM, et al. Association of growth differentiation factor 11/8, putative anti-ageing factor, with cardiovascular outcomes and overall mortality in humans: analysis of the Heart and Soul and HUNT3 cohorts. Eur Heart J. 2015; 36:3426–34. 10.1093/eurheartj/ehv385 26294790 PMC4685178

[21] Egerman MA, Cadena SM, Gilbert JA, Meyer A, Nelson HN, Swalley SE, Mallozzi C, Jacobi C, Jennings LL, Clay I, Laurent G, Ma S, Brachat S, et al. GDF11 increases with age and inhibits skeletal muscle regeneration. Cell Metab. 2015; 22:164–74. 10.1016/j.cmet.2015.05.010 26001423 PMC4497834

[22] Bueno JL, Ynigo M, de Miguel C, Gonzalo-Daganzo RM, Richart A, Vilches C, Regidor C, García-Marco JA, Flores-Ballester E, Cabrera JR. Growth differentiation factor 11 (GDF11) - a promising anti-ageing factor - is highly concentrated in platelets. Vox Sang. 2016; 111:434–36. 10.1111/vox.12438 27509407

[23] Semba RD, Zhang P, Zhu M, Fabbri E, Gonzalez-Freire M, Carlson OD, Moaddel R, Tanaka T, Egan JM, Ferrucci L. Relationship of circulating growth and differentiation factors 8 and 11 and their antagonists as measured using liquid chromatography-tandem mass spectrometry with age and skeletal muscle strength in healthy adults. J Gerontol A Biol Sci Med Sci. 2019; 74:129–36. 10.1093/gerona/gly255 30380014 PMC6298188

[24] Schafer MJ, Atkinson EJ, Vanderboom PM, Kotajarvi B, White TA, Moore MM, Bruce CJ, Greason KL, Suri RM, Khosla S, Miller JD, Bergen HR 3rd, LeBrasseur NK. Quantification of GDF11 and myostatin in human aging and cardiovascular disease. Cell Metab. 2016; 23:1207–15. 10.1016/j.cmet.2016.05.023 27304512 PMC4913514

[25] Poggioli T, Vujic A, Yang P, Macias-Trevino C, Uygur A, Loffredo FS, Pancoast JR, Cho M, Goldstein J, Tandias RM, Gonzalez E, Walker RG, Thompson TB, et al. Circulating growth differentiation factor 11/8 levels decline with age. Circ Res. 2016; 118:29–37. 10.1161/CIRCRESAHA.115.307521 26489925 PMC4748736

[26] Zhou Y, Jiang Z, Harris EC, Reeves J, Chen X, Pazdro R. Circulating concentrations of growth differentiation factor 11 are heritable and correlate with life span. J Gerontol A Biol Sci Med Sci. 2016; 71:1560–63. 10.1093/gerona/glv308 26774117

[27] Mei W, Xiang G, Li Y, Li H, Xiang L, Lu J, Xiang L, Dong J, Liu M. GDF11 protects against endothelial injury and reduces atherosclerotic lesion formation in apolipoprotein E-null mice. Mol Ther. 2016; 24:1926–38. 10.1038/mt.2016.160 27502608 PMC5154476

[28] Fadini GP, Menegazzo L, Bonora BM, Mazzucato M, Persano S, Vigili de Kreutzenberg S, Avogaro A. Effects of age, diabetes, and vascular disease on growth differentiation factor 11: first-in-human study. Diabetes Care. 2015; 38:e118–19. 10.2337/dc15-0868 26116718

[29] Jin M, Song S, Guo L, Jiang T, Lin ZY. Increased serum GDF11 concentration is associated with a high prevalence of osteoporosis in elderly native Chinese women. Clin Exp Pharmacol Physiol. 2016; 43:1145–47. 10.1111/1440-1681.12651 27557752

[30] Freitas-Rodríguez S, Rodríguez F, Folgueras AR. GDF11 administration does not extend lifespan in a mouse model of premature aging. Oncotarget. 2016; 7:55951–56. 10.18632/oncotarget.11096 27507054 PMC5302888

[31] Santanasto AJ, Goodpaster BH, Kritchevsky SB, Miljkovic I, Satterfield S, Schwartz AV, Cummings SR, Boudreau RM, Harris TB, Newman AB. Body Composition Remodeling and Mortality: The Health Aging and Body Composition Study. J Gerontol A Biol Sci Med Sci. 2017; 72:513–19. 10.1093/gerona/glw163 27567109 PMC5897837

[32] Jing YY, Li D, Wu F, Gong LL, Li R. GDF11 does not improve the palmitate induced insulin resistance in C2C12. Eur Rev Med Pharmacol Sci. 2017; 21:1795–802. 28485800

[33] Li H, Li Y, Xiang L, Zhang J, Zhu B, Xiang L, Dong J, Liu M, Xiang G. GDF11 attenuates development of type 2 diabetes via improvement of islet b-cell function and survival. Diabetes. 2017; 66:1914–27. 10.2337/db17-0086 28450417

[34] American Diabetes Association. Classification and diagnosis of diabetes: standards of medical care in diabetes-2019. Diabetes Care. 2019; 42:S13–28. 10.2337/dc19-S002 30559228

[35] Schutz Y, Kyle UU, Pichard C. Fat-free mass index and fat mass index percentiles in Caucasians aged 18-98 y. Int J Obes Relat Metab Disord. 2002; 26:953–60. 10.1038/sj.ijo.0802037 12080449

[36] Gómez-Ambrosi J, Pascual-Corrales E, Catalán V, Rodríguez A, Ramírez B, Romero S, Vila N, Ibáñez P, Margall MA, Silva C, Gil MJ, Salvador J, Frühbeck G. Altered concentrations in dyslipidemia evidence a role for ANGPTL8/betatrophin in lipid metabolism in humans. J Clin Endocrinol Metab. 2016; 101:3803–11. 10.1210/jc.2016-2084 27472196

[37] Angulo P, Keach JC, Batts KP, Lindor KD. Independent predictors of liver fibrosis in patients with nonalcoholic steatohepatitis. Hepatology. 1999; 30:1356–62. 10.1002/hep.510300604 10573511

[38] Gómez-Ambrosi J, Catalán V, Ramírez B, Rodríguez A, Colina I, Silva C, Rotellar F, Mugueta C, Gil MJ, Cienfuegos JA, Salvador J, Frühbeck G. Plasma osteopontin levels and expression in adipose tissue are increased in obesity. J Clin Endocrinol Metab. 2007; 92:3719–27. 10.1210/jc.2007-0349 17595250

[39] Gómez-Ambrosi J, Salvador J, Páramo JA, Orbe J, de Irala J, Diez-Caballero A, Gil MJ, Cienfuegos JA, Frühbeck G. Involvement of leptin in the association between percentage of body fat and cardiovascular risk factors. Clin Biochem. 2002; 35:315–20. 10.1016/S0009-9120(02)00320-X 12135695

